# A rapid HPLC method to simultaneously quantify therapeutic thiols and monitor their disulfide exchange reactions with cystine

**DOI:** 10.1186/s13023-026-04363-w

**Published:** 2026-04-22

**Authors:** Azhidhack Hadjipour, Gayatri Gayatri, Patrice Rioux, Oisín N. Kavanagh

**Affiliations:** 1 School of Pharmacy, Newcastle University, Newcastle upon Tyne, UK; 2 Thiogenesis Therapeutics, San Diego, CA USA

**Keywords:** Disulfide exchange, Cysteamine, Cystinosis, Therapeutic thiols, Cystinuria, Rare disease

## Abstract

This study introduces a rapid and environmentally sustainable High Performance Liquid Chromatography method to simultaneously quantify free thiols and monitor their disulfide exchange kinetics with cystine. This method is based on the derivatisation of thiols with 5,5’-dithiobis(2-nitrobenzoic acid) (DTNB, also known as Ellman’s reagent). The method utilises a mobile phase consisting of 0.1% v/v Trifluoroacetic acid in water and methanol, with Ultraviolet detection at 248 nm. The method exhibited high sensitivity (Limit of Quantification 0.78 µM; Limit of Detection 0.39 µM) and selectivity for biologically relevant thiols (cysteamine, cysteine, tiopronin, glutathione, captopril and penicillamine) in an aqueous environment. Simultaneous quantification of these thiols was successfully achieved in phosphate-buffered saline as well as in biological samples such as human saliva and urine (from a vegetarian subject). Furthermore, it facilitated kinetic analysis of thiol-related reactions, with potential applications in both clinical and pharmacological research.

**Supplementary Information:**

The online version contains supplementary material available at 10.1186/s13023-026-04363-w.

## Introduction

Thiol groups (-SH) are critical functional moieties in pharmaceutical sciences, with both reduced and oxidized forms, such as cysteine and cystine, holding significant biomedical relevance [1]. In conditions such as cystinosis, a rare lysosomal storage disorder, cystine accumulates within lysosomes and precipitates as insoluble crystals, leading to cellular dysfunction and tissue damage. Although cysteamine has been established to enable the depletion of cystine in both laboratory [2] and therapeutic contexts [3–5], we are surprised to find few studies tracking the disulfide exchange of therapeutic thiols on cystine. Much of the early literature explored kinetics with cyclic disulfides [6, 7] and other molecules of biological interest, like glutathione, with the view to develop structure activity relationships [8]. Just one study examined penicillamine disulfide exchange with cystine [9]. Whitesides has published an excellent comprehensive review of this work [10]. 

**Fig. 1:**
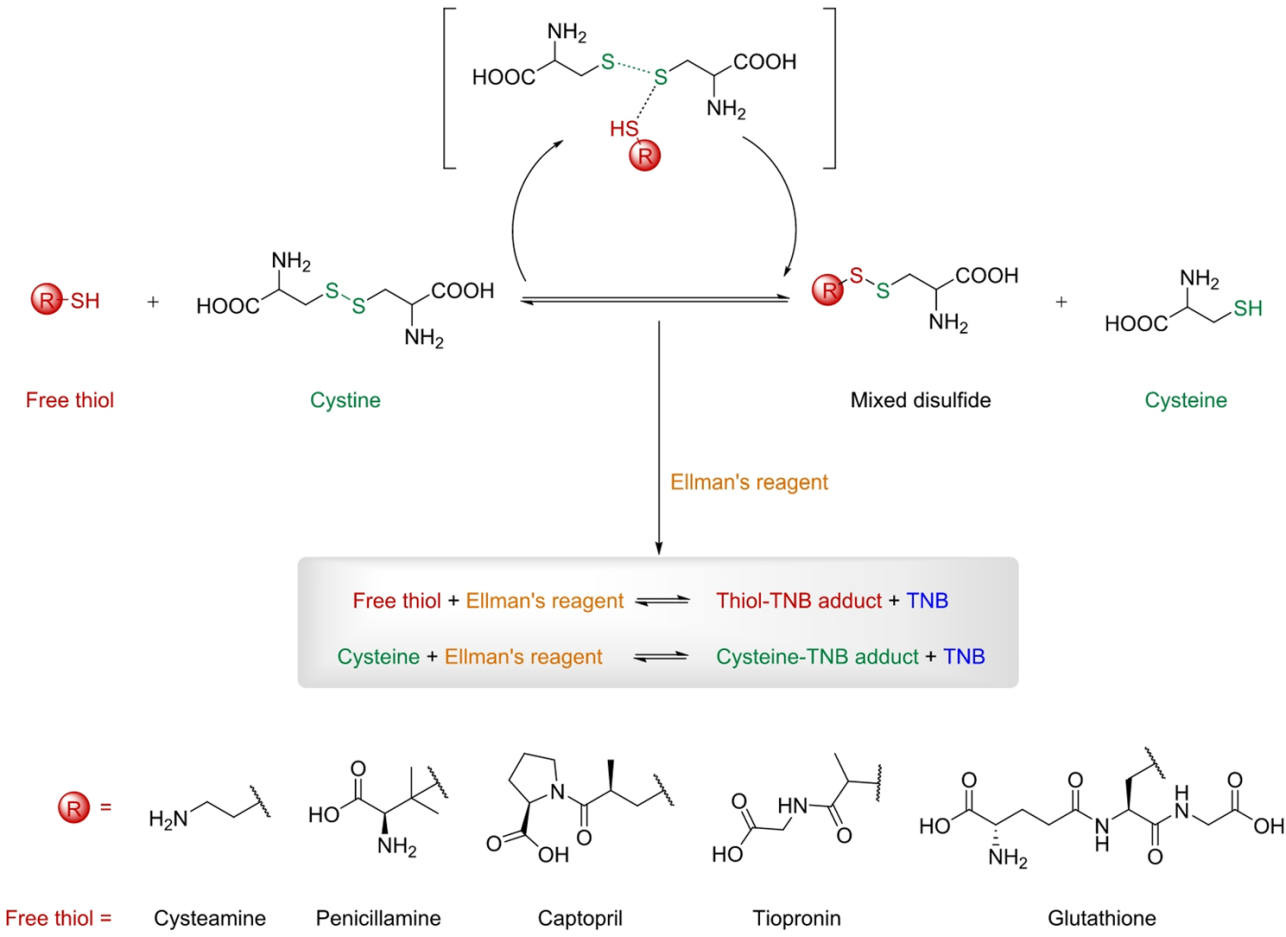
General disulfide exchange mechanism of cystine in the presence of therapeutic free thiols examined in this study

To monitor this important reaction we set out to develop a method which can quantify and distinguish the various free thiols in solution during a reaction. Although several methodologies have been documented in the literature, they are often associated with practical drawbacks [11]. For instance, extended retention times may pose challenges due to the increased susceptibility of free thiols to oxidative degradation (Table 1). Additionally, the prevalent use of acetonitrile as a mobile phase solvent, despite its effectiveness, raises environmental and safety concerns, given its classification as a non-green solvent [12]. In the present study, we report the development and validation of a rapid, cost-effective, and environmentally sustainable high performance liquid chromatography (HPLC) method for the quantification of free thiols (Fig. 1). The method has been optimised for high-throughput analysis, requiring minimal reagent consumption and offering short run times. Crucially, this method was successfully utilised to monitor the reduction of cystine by various biologically relevant free thiol-containing compounds, including cysteamine, under physiologically relevant conditions, thereby enabling the kinetic analysis of these therapeutically important redox reactions.

**Table 1:** Table represents a brief comparison of the existing free thiol detection methods vs. the presented method in this study

Method	Sensitivity	Runtime	Environmental Impact	Advantages	Limitations
HPLC (presented in this paper)	nM-µM	Short (≤ 20 min)	Moderate (minimal solvent, moving away from Acetonitrile as a non-green solvent)	Simple, rapid, minimal waste, Selectivity, Simultaneous detection/quantification. Highly reduces the potential sample oxidation due to the lower runtime. Cost effective due to minimal solvent	Lower sensitivity versus highly sensitive methods. Sample pre-treatment in some biological samples (non-vegetarian)
Fluorescence labelling (e.g., monobromobimane)	nM–µM	Medium (derivatisation + HPLC, e.g., ~ 20–40 min)	Moderate (organic solvents, fluorescent reagents)	Very high sensitivity and selectivity	Extra steps and reagents, longer prep time, risk of sample oxidation due to longer runtime
Ellman’s reagent (DTNB) UV-vis	nM-µM	Short–medium (colorimetric assay or pre-column derivatisation)	Moderate (reagent use, some waste)	Rapid and widely used; stoichiometric reaction; easy setup)	No Selectivity, Less sensitive than fluorescent methods
High-sensitivity (e.g., MS-based, LIF-CE)	pM–nM	Variable	High (specialized solvents and consumables)	Exceptional sensitivity and structural data	Expensive, complex setup, poor sustainability

## Materials & methods

HPLC grade acetonitrile, methanol, and water were purchased from Scientific Laboratory Supplies. Ellman’s reagent was purchased from Sigma-Aldrich and all other materials were purchased from Tokyo Chemical Industries at the highest purity available.

### Ellman’s assay

A 1.25 mM solution of 5,5’-dithiobis-(2-nitrobenzoic acid) (DTNB) was prepared in a 50:50 methanol (MeOH): H_2_O mixture and stored in darkness. To each 200 µL experimental aliquot, 200 µL of the DTNB stock was added and adjusted to a final volume of 4 mL by the addition of 3.6 mL of phosphate-buffered saline (PBS) (0.1 M, pH 8.0), with pH of 8 being critical to ensure efficient derivatisation of thiol groups. The reaction mixtures were then incubated at ambient temperature for 15 min to allow complete reaction between the free thiol groups and DTNB. Following incubation, 3 mL of the derivatised solution was transferred into a quartz cuvette for UV spectrophotometric or chromatographic analysis. Care was taken to ensure that an excess of DTNB was maintained in all reactions to drive complete conversion and ensure accurate quantification. For UV-Vis, derivatisation of the free thiol produced a prominent absorbance peak at 412 nm, indicative of 5-thio-2-nitrobenzoic acid (TNB). For reference, DTNB $\:\lambda\:$_max_ = 323 nm (Fig. S1).

### High-performance liquid chromatography (HPLC) method development

A wide-spectrum analysis (200–450 nm) of a derivatised 0.05 mM cysteine sample was conducted using a DAD-equipped Agilent 1260 Infinity HPLC system to identify the optimum detection wavelength. Separation was performed on a Kromasil 100-5-C18 (4.6 × 250 mm) column at a flow rate of 1 mL/min, with the column temperature maintained at 20 °C, over a 20-minute run. Based on the area under the curve (AUC) values and their variation across different wavelengths, 248 nm was selected as the optimal wavelength for peak detection from the region with the closest range of values for all peaks (Fig. S2). The mobile phases employed were 0.1% Trifluoroacetic acid (TFA) in water and MeOH across a range of methods (Table 2) to identify a method for simultaneous resolution of all analytes, namely TNB, the thiol-TNB adduct, and DTNB. For mobile phase optimisation, a derivatised 0.05 mM cysteine sample was analysed utilizing various mobile phase ratios (from 30:70 to 70:30) of both 0.1% TFA in water-methanol (MeOH) and 0.1% TFA in water-acetonitrile (ACN), employing both isocratic and gradient conditions. The analytical parameters were set at a detection wavelength of 248 nm, a column temperature of 20 °C, an injection volume of 10 µL, and a flow rate of 1 mL/min.

**Table 2:** Preliminary high performance liquid chromatography method development with comment on suitability. Mobile phase optimisation with detection wavelength = 248 nm, T = 20 °C, injection volume = 10 µL, and flow rate = 1 mL/min

Method	Comment	Advantage
Gradient, 30:70 to 70:30 over 20 min, 0.1% TFA: MeOH	Unacceptable, Within/between-run baseline variation.	-
Isocratic, ≤ 35:65, 0.1% TFA: MeOH	Unacceptable, Overlapping peaks	-
Isocratic, 40:60, 0.1% TFA: MeOH	Acceptable	Lower Rt
Isocratic, 45:55, 0.1% TFA: MeOH	Acceptable	Higher Rs
Isocratic, 50:50, 0.1% TFA: MeOH	Unacceptable, High Rt	-
Isocratic, 55:45, 0.1% TFA: MeOH	Unacceptable, High Rt	-
Isocratic, 60:40, 0.1% TFA: MeOH	Unacceptable, High Rt	-
Isocratic, ≤ 45:55, 0.1% TFA: ACN	Unacceptable, Overlapping peaks	-
Isocratic, 50:50, 0.1% TFA: ACN	Acceptable	Lower Rt
Isocratic, 55:45, 0.1% TFA: ACN	Acceptable	Higher Rs
Isocratic, 60:40, 0.1% TFA: ACN	Acceptable	Higher Rs
Isocratic, 65:35, 0.1% TFA: ACN	Unacceptable, High Rt	-

### Robustness

Robustness of the developed HPLC methods was systematically evaluated to determine their resilience to minor, deliberate variations in critical analytical parameters. Specifically, the effects of column temperature fluctuations (± 2 °C), mobile phase stability following two weeks of storage, the impact of flow rate changes (0.98–1.02; ± 2% of nominal) and slight pH changes due to the process involved for preparation of 0.1% TFA as mobile phase preparation phase by the operator. Furthermore, the influence of injection volume variation on detector response was assessed by reducing the injection volume by 50%. The results demonstrated no significant differences in retention time, peak resolution or peak area for the TNB analytes under these modified conditions, indicating that the method performance remains consistent. This analysis enabled us to arrive at final methods of 40:60 or 45:55, 0.1% TFA: MeOH with isocratic flow at 1 mL/min with the column temperature maintained at 20 °C.

### Stability testing

Three independent replicates of derivatised cysteine (0.05 mM) and cysteamine (0.05 mM) were prepared. Subsequently, 1.5 mL of each sample was transferred into an HPLC vial and labelled according to the storage conditions: ambient temperature, freezer at -20 °C, and refrigerator at 2–8 °C. The samples were immediately analysed using the optimized methods, with the free thiol concentration recorded at time point T0 for each sample. Following this, the samples were placed under their respective storage conditions and analysed at predetermined intervals: 12 h (T12), 24 h (T24), and 48 h (T48). Sample preparation and data analysis in all cases were performed in triplicate to ensure methodological reproducibility and data consistency.

### Sample preparation and analysis in PBS

To 300 µL of a 5 mM solution of Ellman’s reagent, 300 µL of a mixed thiol solution, comprising 50 µL aliquots from each of the six thiol stock solutions, was added. The total volume was adjusted to 4 mL with PBS (pH 8.0). Subsequently, 1.5 mL of this reaction mixture was transferred to an HPLC vial and analysed using the 45:55 method. Sample preparation and data analysis in all cases were performed in triplicate to ensure methodological reproducibility and data consistency.

### Sample preparation and analysis in human saliva and urine

Stock solutions (4 mM) of six biologically relevant free thiols, namely cysteine, cysteamine, penicillamine, glutathione, tiopronin, and captopril were individually prepared in PBS at pH 8.0. Human saliva and urine samples were freshly collected from two volunteers (non-fasting at midday) adhering to distinct dietary habits, namely vegetarian and non-vegetarian, to assess potential biological variability. The collection of urinary samples followed the mid-stream collection technique to minimize contamination from urethral and external sources. A 50 µL aliquot from each of the six thiol stock solutions was added to 300 µL of either saliva or urine. This mixture was immediately treated with 300 µL of 5 mM Ellman’s reagent and brought to a final volume of 4 mL with PBS (pH 8.0). Following thorough mixing, 1.5 mL of this mixture was transferred to an HPLC vial for analysis under the same chromatographic conditions (45:55 method). All experiments were conducted in triplicate for both biological matrices (saliva and urine) and dietary groups to ensure methodological reproducibility and to assess inter-individual variability. This project was granted project ethical approval (59267/2023).

### Investigation of disulfide exchange kinetics

Free thiol and cystine solutions (0.5 mM) were individually prepared in PBS (pH 8.0). To dissolve the sparingly soluble cystine at ambient temperature, the solution was heated to 60 °C for 20 min, followed by an additional 60 min of stirring at ambient temperature; this is below the equilibrium solubility [1]. Subsequently, the prepared 0.5 mM cystine solution was filtered using a 0.45 μm syringe filter and a 10 mL aliquot was mixed with an equal volume of the pre-prepared 0.5 mM cysteamine solution. The resulting 20 mL solution contained 0.25 mM of each cysteamine and cystine in PBS (pH 8.0). The reaction vessel was immediately sealed and continuously stirred by a magnetic stirrer throughout the duration of the experiment.

To monitor the first 5 min of the reaction, at each predefined time interval (5, 30, 60, 90, 120, 150, 180, 210, 240, 270, 300 s), 500 µL of the reaction solution was withdrawn and mixed with 200 µL of the 1.25 mM Ellman’s reagent solution (in excess relative to free thiol groups), effectively quenching the reaction between cysteamine and cystine. This mixture was then supplemented with an additional 3.3 mL of PBS (pH 8.0), resulting in a solution that contains 0.03125 mM cysteamine and 0.0625 mM Ellman’s reagent at t = 0 s, corresponding to a molar ratio of 1:2 (Cysteamine: Ellman’s reagent). A 1.5 mL aliquot of the derivatised solution was subsequently transferred to an HPLC vial and analysed using the 45:55 (0.1% TFA: MeOH) method. The same method was applied to all free thiol-containing compounds.

## Results & discussion

### High-performance liquid chromatography (HPLC) method development

A detection wavelength of 248 nm was selected based on preliminary spectral analysis (Fig. S1 and S2). Subsequently, optimisation of the mobile phase composition was undertaken (Table 2), employing both isocratic and gradient elution profiles ranging from 70:30 to 30:70 ratios of 0.1% Trifluoroacetic acid (TFA) in water to methanol (MeOH) and acetonitrile (ACN), respectively. We found that increasing the proportion of methanol in the mobile phase led to a reduction in retention time (R_t_) for the three analytes, albeit accompanied by a concomitant decrease in peak resolution. To systematically evaluate this effect, a comparative summary of three isocratic methods utilizing distinct mobile phase ratios of 0.1% TFA in water to methanol was compiled (Table 3). This table reports the retention times of 5,5’-dithiobis-(2-nitrobenzoic acid) (DTNB), along with the resolution values between the 5-thio-2-nitrobenzoic acid (TNB) and thiol-TNB adduct peaks under each condition. The mobile phase compositions of 45:55 and 40:60 (0.1% TFA in water: MeOH) were selected for full characterization, as they represent more environmentally sustainable and efficient conditions.

**Table 3:** Table presents retention times of DTNB (also known as Ellman’s reagent) and the calculated resolutions of TNB to thiol-TNB adducts for a derivatised 0.05 mM solution of free thiol (cysteamine) at varying mobile phase ratios

Method TFA 0.1% in water: MeOH	Rt (mins, DTNB)	Resolution (TNB to thiol-TNB adduct)
40:60	10.64	13.67
45:55	16.82	16.26
50:50	30.48	18.51

### Linearity

The linearity of the HPLC-developed methods, employing mobile phase compositions of 40:60 and 45:55 (0.1% TFA: MeOH), was thoroughly assessed for six thiol-containing compounds, namely cysteine, cysteamine, penicillamine, glutathione, tiopronin, and captopril. Calibration curves were constructed for each thiol over the concentration range of 6–60 µM. The results demonstrated a high degree of linearity, with R² values consistently ranging from 0.999 to 1.0, confirming the robustness and precision of the method (*n* = 3). This strong linear relationship between analyte concentration and detector response across the studied concentration range indicates that the HPLC methods are highly suitable for accurate quantification of these thiols, ensuring reliable performance for analytical applications.

### HPLC method precision

The within-run precision for each optimised method was determined by performing six consecutive injections of the same derivatised sample, specifically 0.05 mM cysteine and 0.05 mM cysteamine. The retention times of TNB, thiol-TNB adducts, and the free thiol concentrations were compared across six distinct runs for each sample. The between-run precision for each optimized method was assessed by preparing six separately derivatised samples of 0.05 mM cysteine and cysteamine on different days. These prepared samples were subsequently analysed at different intervals, and the retention times of TNB, thiol-TNB adducts, and the free thiol concentrations were compared across six independent runs for each thiol (Table 4 & S4). The results demonstrate acceptable precision in both retention times (Rt) and free thiol concentrations, with consistent performance observed across both inter-run and intra-run measurements.

**Table 4:** Precision analysis for both within-run and between-run variability for derivatised 0.05 mM samples of cysteamine and cysteine. The data, presented as mean ± standard deviation (S.D.), include the retention times of thiol-TNB adducts, retention times of TNB peaks, and concentrations of free thiols

Thiol	Thiol-TNB adduct Rt (mins) Mean ± S.D. (%RSD)		TNB Rt (mins) Mean ± S.D. (%RSD)	Free Thiol (mM) Mean ± S.D. (%RSD)
**Within-run (same vial)**				
Cysteamine	3.03 ± 0.0021 (0.07)		4.44 ± 0.0033 (0.07)	0.05 ± 0.0003 (0.6)
Cysteine	2.93 ± 0.0016 (0.05)		4.44 ± 0.0033 (0.07)	0.05 ± 0.0001 (0.2)
**Between-runs (different vials)**				
Cysteamine	3.03 ± 0.0204 (0.67)		4.41 ± 0.0262 (0.59)	0.05 ± 0.0004 (0.8)
Cysteine	2.91 ± 0.0169 (0.58)		4.41 ± 0.0262 (0.59)	0.05 ± 0.0002 (0.4)

### Selectivity

**Fig. 2:**
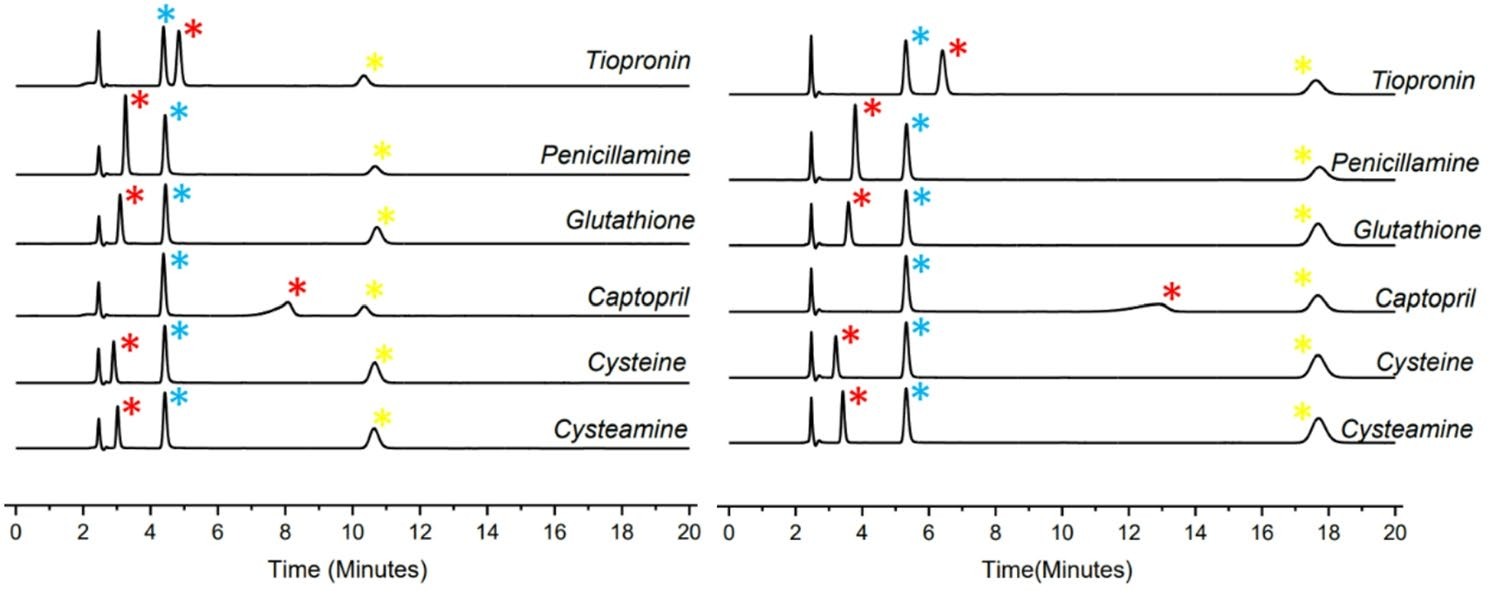
Representative set of chromatograms (0.05 mM derivatised solution of free thiols) for the 40:60 (0.1% Trifluoroacetic acid: Methanol) method (left) and 45:55 (0.1% Trifluoroacetic acid: Methanol) method (right). Thiol-TNB adducts, TNB, and Ellman’s reagent peaks are indicated by red, blue, and yellow asterisk, respectively

The findings indicate that both methods effectively differentiate between the TNB adducts of the different thiols analysed, with one method offering superior resolution, while the other provides reduced retention times and shorter overall run duration (Table 5; Fig. 2). These results underscore the capacity of the optimized methods to distinguish a diverse range of thiol species within a rapid and efficient analytical framework.

**Table 5:** Physicochemical properties of selected free thiols and their retention times (R_t_) across two methods (*n* = 3)

Thiol	Calc. Log *P*	Molecular weight (g/mol)	Calc. pK_a_	40:60 TNB adduct Rt (mins) Mean ± S.D.	45:55 TNB adduct Rt (mins) Mean ± S.D.
Cysteine	-2.80	121.16	2.35, 9.05	2.91 ± 0.0169	3.20 ± 0.0213
Cysteamine	-0.42	77.15	9.42, 10.40	3.03 ± 0.0204	3.38 ± 0.0356
Penicillamine	-2.10	149.21	2.56, 9.09	3.26 ± 0.0114	3.79 ± 0.0125
Captopril	0.73	217.29	-1.30, 4.02	8.06 ± 0.0117	12.89 ± 0.0207
Tiopronin	-0.53	163.19	-4.20, 3.86	4.84 ± 0.0128	6.42 ± 0.0157
Glutathione	-4.90	307.32	1.94, 9.22	3.09 ± 0.0122	3.59 ± 0.0225

### Sensitivity

The limit of detection (LOD) for both ultraviolet-visible (UV-Vis) and the optimized HPLC methods was determined using serial dilutions of derivatised free thiol standards, namely cysteine and cysteamine, down to the lowest concentration (signal-to-noise (S/N) = 3:1) at which a detectable peak was observed (Fig. S3 & S6). The limit of Quantification (LOQ) for both UV-Vis and the optimized HPLC methods was determined using serial dilutions of derivatised free thiol standards, namely cysteine and cysteamine, down to the lowest concentration (S/*N* = 10:1) at which a detectable peak could be accurately quantified with acceptable precision and accuracy. A summary table comprising the corresponding free thiol concentrations and percentage recoveries is presented below (Table 6 & S5). The results indicate that a concentration of 0.78 µM represents the limit of quantification (LOQ) for both analytical techniques, with percentage recovery values falling within an acceptable range, thereby highlighting the sensitivity and reliability of the methods.

**Table 6:** The limit of quantification data presented as percentage recovery (Mean ± S.D.) for derivatized cysteine and cysteamine samples. Analyses were performed using the optimized 40:60 (0.1% Trifluoroacetic acid: Methanol) HPLC method (*n* = 6). The data reflect the sensitivity and reproducibility of the method at low analyte concentrations

**UV-vis**			
**Cysteine (mM)**	**Absorbance (A.U.)** **Mean ± S.D.**	**Calculated Thiol (mM)**	**Recovery (%)** **Mean ± S.D.**
0.00625	0.0856 ± 0.0045	0.006244	99.90 ± 4.33
0.003125	0.0434 ± 0.0019	0.003167	101.35 ± 4.5
0.0015625	0.0217 ± 0.0013	0.001588	101.63 ± 6.02
0.00078125	0.0109 ± 0.0003	0.000800*	102.40 ± 2.94
**HPLC**			
**Cysteamine (mM)**	**TNB Rt (mins)**	**Calculated Thiol (mM)**	**Recovery (%)** **Mean ± S.D.**
0.00625	4.40 ± 0.004	0.006246	99.94 ± 2.82
0.003125	4.40 ± 0.004	0.003071	98.27 ± 1.63
0.0015625	4.40 ± 0.004	0.001602	102.53 ± 1.35
0.00078125	4.40 ± 0.004	0.000796*	101.89 ± 2.50
**Cysteine (mM)**	**TNB Rt (mins)**	**Calculated Thiol (mM)**	**Recovery (%)** **Mean ± S.D.**
0.00625	4.40 ± 0.004	0.006247	99.96 ± 1.54
0.003125	4.40 ± 0.004	0.003110	99.52 ± 2.21
0.0015625	4.40 ± 0.004	0.001588	101.63 ± 2.08
0.00078125	4.40 ± 0.004	0.000795*	101.76 ± 2.97

Mean ± S.D.* Limit of quantification (LOQ) in acceptable recovery range

**Fig. 3:**
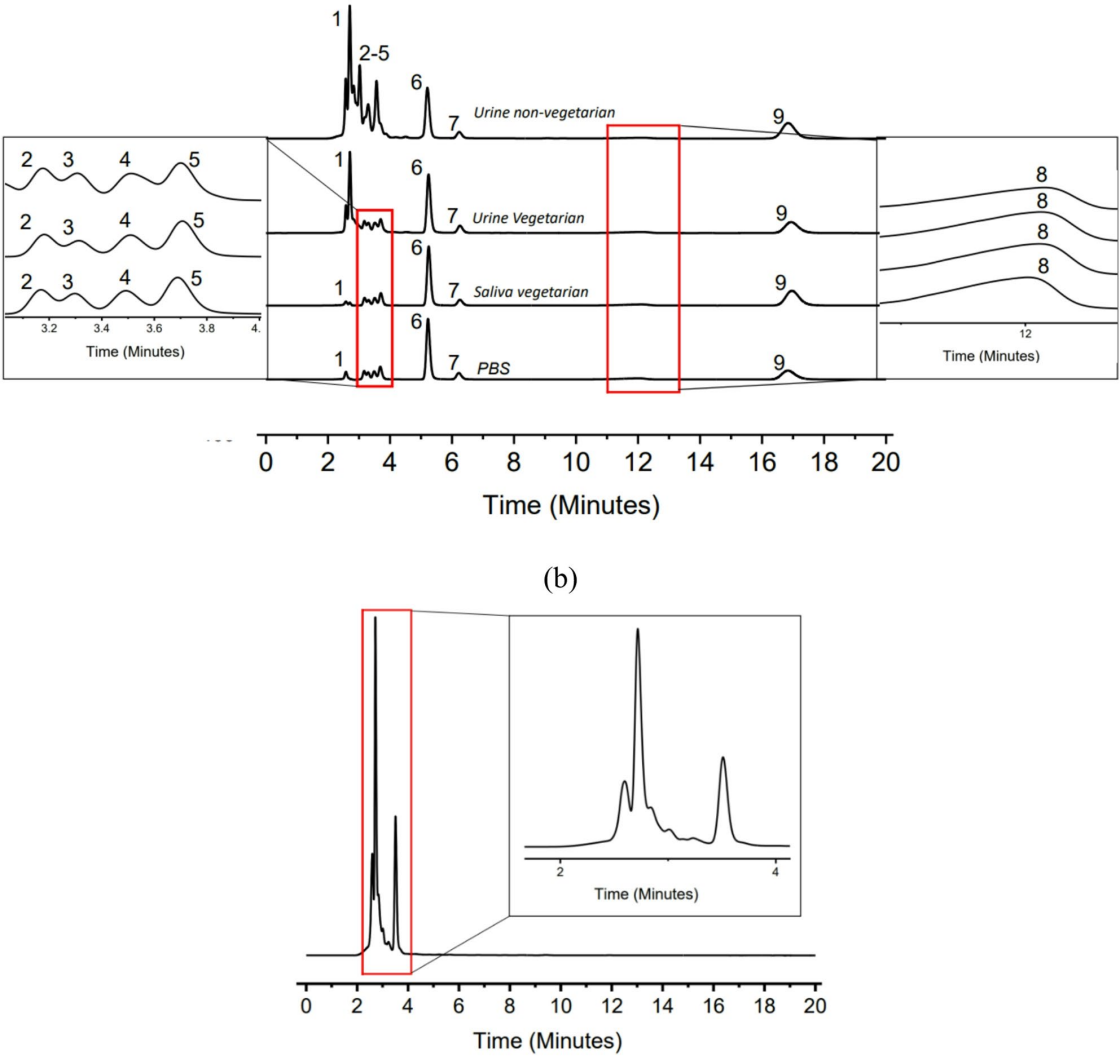
Graph (**a**) represents detection of derivatised free thiols in phosphate-buffered saline, saliva and urine (human vegetarian sample), using HPLC 45:55 (0.1% Trifluoroacetic acid: Methanol) method. (1) Blank, (2) Cysteine-TNB adduct, (3) Cysteamine-TNB adduct, (4) Glutathione-TNB adduct, (5) Penicillamine-TNB adduct, (6) TNB, (7) Tiopronin-TNB adduct, (8) Captopril-TNB adduct, (9) Excess Ellman’s reagent (*n* = 3). Note that no back ground noise was detected in vegetarian (saliva & urine) samples in the region of interest. Resolution of peaks 2–5 was not achieved in the non-vegetarian non-fasted sample due to the background noises in the region of interest. Graph (**b**) represents the urine non-vegetarian non-fasted chromatogram before free thiol incorporation, illustrating background noise in the region of peaks 2–5

Using the 45:55 (0.1% TFA: MeOH) HPLC method, six biologically relevant thiols, namely cysteine, cysteamine, penicillamine, glutathione, tiopronin, and captopril were successfully determined and quantified in phosphate-buffered saline (PBS, pH 8.0), human saliva and urine (from a human vegetarian subject) (Fig. 3 and Table S1). However, quantification of thiols in a non-fasted non-vegetarian subject was not achieved due to significant background noise in the chromatographic region of interest. To improve the clarity of chromatograms and reduce background interference, it may be advisable to collect biological samples after a period of vegetarian diet and adequate hydration. Additionally improve the sample clarification (Table 7). All of these analysis were carried out in triplicate to ensure the accuracy and reproducibility of data. Table 7 represents a brief overview of the method application on complex biological fluids, including Limitations of Using the Method Directly on Complex Biological Fluids and proposed mitigation strategies. 

**Table 7:** Summary table representing a brief overview of the method application on complex biological fluids, including limitations of using the method directly on complex biological fluids and proposed mitigation strategies

Category	Details
Limitations of Using the Method Directly on Complex Biological Fluids	• Matrix Effects & Background Interference: Complex fluids contain proteins, peptides, metabolites, and dietary compounds that can absorb near the same wavelength as free thiol TNB-adducts or co-elute with derivatised thiols, reducing resolution and accuracy. • Variable Thiol Oxidation State: Free thiols can oxidize to disulfides during collection or processing, underestimating true concentrations unless reduced before derivatisation. • Stability of TNB Adducts: DTNB reaction products may degrade or react with other nucleophiles if not analysed promptly, introducing variability. • Dietary and Lifestyle Variability: Amino acid intake, supplements, and hydration can alter sample composition and baseline noise. • Limit of Detection in Noisy Backgrounds: High baseline absorbance can reduce sensitivity, especially for rare disease applications.
Implications for Clinical Applications in Rare Diseases	• Poor resolution may cause false negatives or positives in diagnostic applications. • Dietary variability complicates reproducibility across sites or over time.
Mitigation Strategies	• Pre-collection controls: Overnight fasting (8–12 h for urine; 1–2 h for saliva) and avoiding sulfur-rich foods and adequate hydration reduce background interference. • Immediate sample handling: Keep samples on ice, adjust pH to 7.5–8.0, and process within 30–60 min. • Sample clarification: Centrifugation, filtration, or ultrafiltration reduce matrix complexity.
Supporting Points for the Method	• Simultaneous detection of multiple low-molecular-weight thiols in one injection. • Medium–high sensitivity and reproducible selectivity for TNB-adducts across sample types. • Rapid analysis minimizes oxidation of free thiols, preserving accurate quantification. • Rapid analysis enabling same-day clinical turnaround. • Greener chemistry using methanol instead of acetonitrile. • Technician-friendly with standard RP columns and simple derivatisation. • DAD spectral fingerprints of TNB-adducts allow identity confirmation without MS.

### Recovery

Three independent derivatised solutions of free thiol (cysteamine) at known concentrations were prepared in PBS pH 8.0 and analysed using the optimized analytical methods. Each solution was measured in triplicate to assess the method’s reproducibility. The results comprising obtained concentrations and percentage recoveries are summarized in (Table 8 & S3). The data demonstrate acceptable percentage recoveries and low standard deviations, supporting the reliability and reproducibility of the developed methods.

**Table 8:** Table presents the percentage recovery data (Mean ± S.D.) for three independently prepared solutions of the free thiol-containing compound, cysteamine, at known concentrations. Analyses were performed using the optimized 40:60 (0.1% Trifluoroacetic acid: Methanol) HPLC method. The results indicate acceptable recovery rates with minimal standard deviations, thereby affirming the reliability, precision, and reproducibility of the developed analytical method

Samples	Theoretical (mM)	Obtained (mM) R1	Obtained (mM) R2	Obtained (mM) R3	Obtained (mM) Mean ± S.D.	Recovery (%) Mean ± S.D.
I	0.0112	0.0109	0.0108	0.0107	0.0108 ± 0.0001	96.43 ± 0.91
II	0.0204	0.0206	0.0201	0.0200	0.0202 ± 0.0003	99.51 ± 1.39
III	0.0389	0.0386	0.0383	0.0386	0.0385 ± 0.0001	98.97 ± 0.36

### Stability

**Fig. 4:**
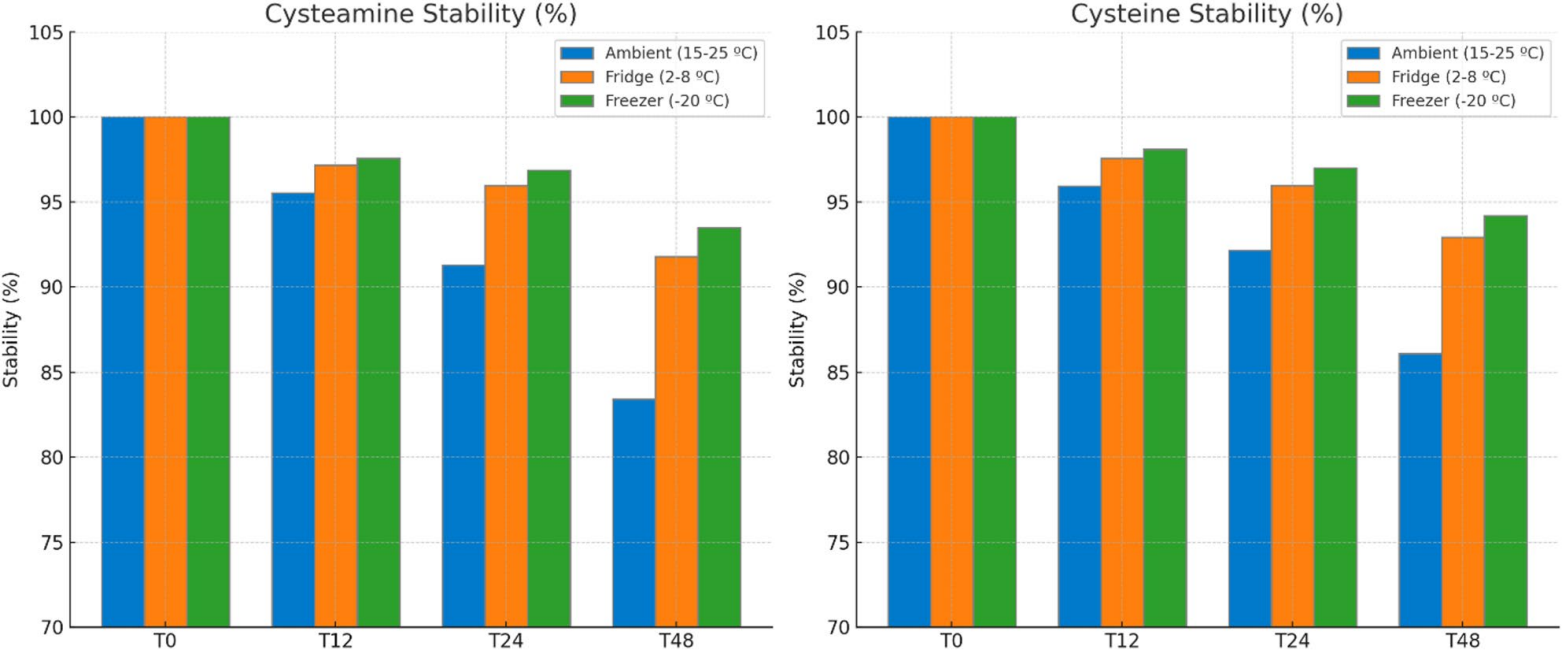
Representation of stability data for 0.05 mM TNB as a % of control (T0) over 48 h period at different storage conditions using 40:60 (0.1% Trifluoroacetic acid: Methanol) method

To evaluate the practical limitations of the optimised analytical methods with respect to storage conditions, free thiol standards, namely cysteine and cysteamine, were analysed under controlled storage environments at predetermined time intervals. The percentage recoveries of the derivatised free thiols throughout the duration of the study are presented in a summary table (Table 9 & S2), accompanied by graphical representations illustrating the degradation profiles over time (Fig. 4, S4 & S5). As expected, the data clearly demonstrate the superior stability of free thiol-Ellman’s adducts when stored under refrigerated and frozen conditions, as compared to ambient temperature. Notably, both refrigeration and freezing exhibited comparable recovery profiles over the 48-hour experimental period, supporting their suitability for short-term preservation of thiol-containing samples.

**Table 9:** Stability data for 0.05 mM TNB as a % of control (T0) over 48 h period at different storage conditions using 40:60 (0.1% Trifluoroacetic acid: Methanol) method

**Cysteamine** **0.05 mM**	**Ambient (15–25 °C)** **Mean ± S.D.** **(%RSD)**	**Fridge (2–8 °C)** **Mean ± S.D.** **(%RSD)**	**Freezer (-20 °C)** **Mean ± S.D.** **(%RSD)**
T0	100.00 ± 0.0 (0.0)	100.00 ± 0.0 (0.0)	100.00 ± 0.0 (0.0)
T12	95.53 ± 0.51 (0.53)	97.16 ± 0.24 (0.25)	97.55 ± 0.20 (0.21)
T24	91.29 ± 0.68 (0.74)	95.97 ± 0.36 (0.38)	96.85 ± 0.29 (0.30)
T48	83.41 ± 0.88 (1.06)	91.79 ± 0.53 (0.58)	93.48 ± 0.47 (0.50)
**Cysteine** **0.05 mM**	**Ambient (15–25 °C)** **Mean ± S.D.** **(%RSD)**	**Fridge (2–8 °C)** **Mean ± S.D.** **(%RSD)**	**Freezer (-20 °C)** **Mean ± S.D.** **(%RSD)**
T0	100.00 ± 0.0 (0.0)	100.00 ± 0.0 (0.0)	100.00 ± 0.0 (0.0)
T12	95.94 ± 0.34 (0.35)	97.55 ± 0.23 (0.24)	98.10 ± 0.17 (0.17)
T24	92.14 ± 0.61 (0.66)	95.97 ± 0.37 (0.39)	96.99 ± 0.31 (0.32)
T48	86.08 ± 0.76 (0.88)	92.90 ± 0.45 (0.48)	94.20 ± 0.39 (0.41)

### Application to disulfide exchange kinetics

**Fig. 5:**
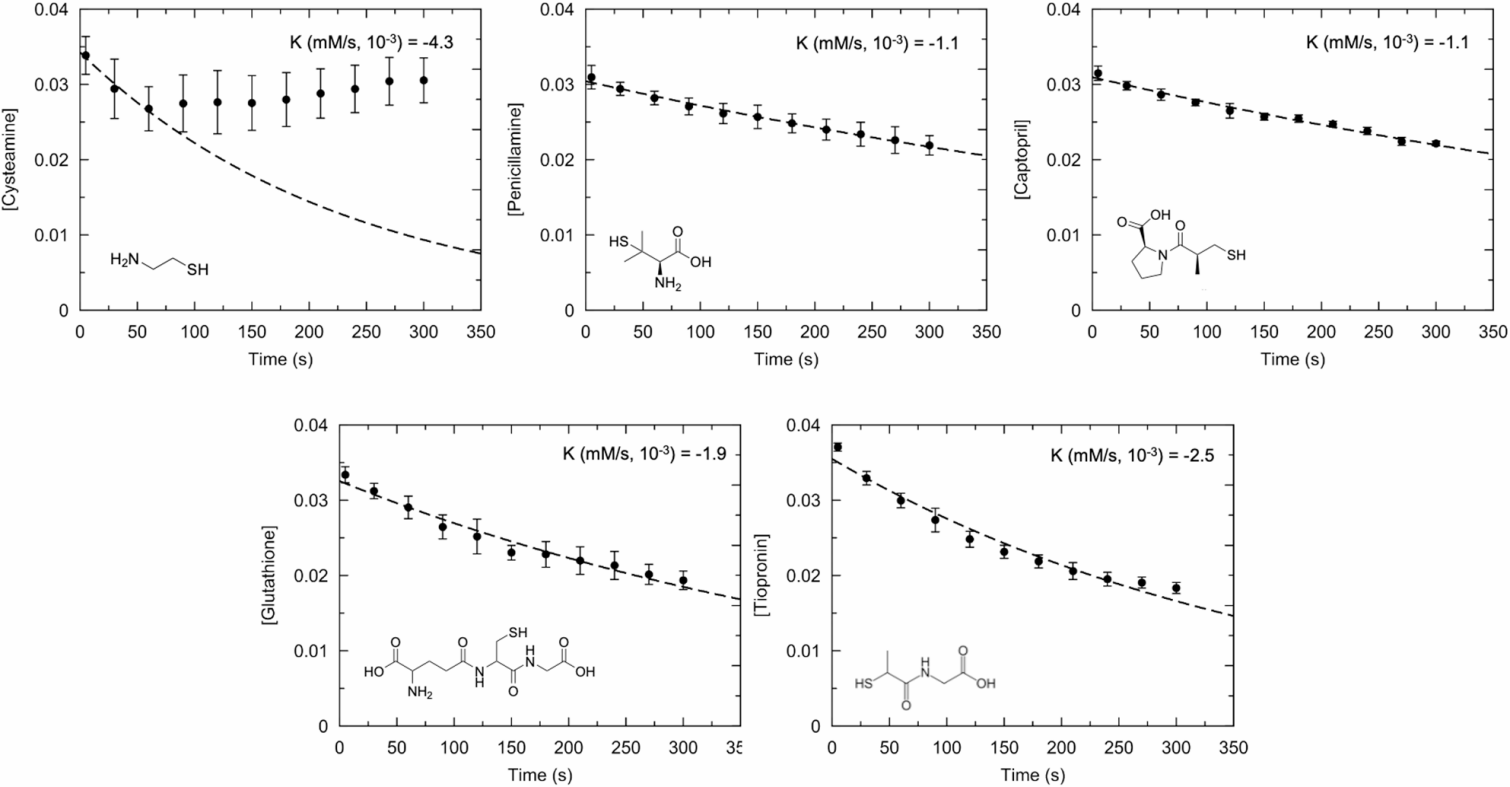
Consumption kinetics for a variety of free thiols (starting concentration = 0.03125 mM) in the presence of cystine (starting concentration 0.03125 mM), monitored by Ellman’s reagent (starting concentration = 0.0625 mM)

We applied this method to monitor the disulfide exchange reaction between free thiols and cystine via simultaneous quantification of the various free thiols involved in this reaction. This is an important reaction to evaluate the efficacy of disulfide reducing therapeutics (Fig. 1). The general rate of thiol disulfide exchange has been found to be overall second order, but with each reaction first-order [10]. The temporal profile of most thiols follows an apparent first-order rate constant, where the apparent rates for free thiol consumption in the presence of cystine is summarised in Fig. 5, we attempted crude correlation with logP, molecular weight and solubility; however, we do not find any significant trends. We observe anomalous behaviour for cysteamine and cysteine TNB adducts which appear to be increasing in concentration while TNB and Ellman’s are decreasing (Fig. S7-S9). All other thiol TNB adducts are stable for 48 h, such that the determined concentrations during the kinetics experiments reflect the concentration in the solution at that time. We cannot be sure what the concentration of cysteamine or cysteine is in the kinetics experiments due to progressive drift of their TNB adducts towards the maximum theoretical concentrations (0.03125 and 0.0625 mM respectively). We suspect that either the cysteamine-cysteine adduct is unstable or that the residual cystine produces more cysteine, reacting with the remaining Ellman’s. This hypothesis is supported by the observation that cysteine-TNB adduct concentrations after 24 h reach the maximum theoretical concentration of 0.0625 mM. This is complex as multiple equilibrium reactions are at play. It is worth to mention that this is a preliminary step in this regard and require further investigation.

## Conclusion

In this study, a rapid, efficient, and environmentally sustainable high performance liquid chromatography method has been developed for the quantification of free thiols in aqueous solutions. This method facilitates the precise analytical determination of a broad spectrum of free thiol species, meeting pharmacopeial standards. Furthermore, it has successfully enabled the simultaneous quantification of several biologically relevant thiols in human saliva and urine samples. Additionally, the method was employed to monitor the reaction kinetics between free thiols and cystine, a reaction of both mechanistic and clinical significance.

## Supplementary Information

Below is the link to the electronic supplementary material.

Supplementary Material 1

## Data Availability

Data is available on request fix style

## References

[1] Noble K, Kavanagh N. Cystine Crystal Nucleation and Decay in the Context of Cystinuria Pathogenesis and Treatment. RSC Adv. 2024;14(44):32063–72. 10.1039/D4RA04469J. 39391615 10.1039/d4ra04469j PMC11465997

[2] Thoene JG, Oshima RG, Crawhall JC, Olson DL, Schneider JA. Cystinosis. Intracellular Cystine Depletion by Aminothiols in Vitro and in Vivo. J Clin Invest. 1976;58(1):180–9. 10.1172/JCI108448. 932205 10.1172/JCI108448 PMC333169

[3] Gahl WA, Ingelfinger J, Mohan P, Bernardini I, Hyman PE, Tangerman A. Intravenous cysteamine therapy for nephropathic cystinosis. 1995;38(4). 10.1203/00006450-199510000-00018 8559613

[4] Jones NP, Postlethwaite RJ, Noble JL. Clearance of Corneal Crystals in Nephropathic Cystinosis by Topical Cysteamine 0.5%. Br J Ophthalmol. 1991;75(5):311–2. 10.1136/bjo.75.5.311. 2036352 10.1136/bjo.75.5.311 PMC1042364

[5] Levtchenko EN, van Dael CM, de Graaf-Hess AC, Wilmer MJG, van den Heuvel LP, Monnens LA, Blom HJ. Strict cysteamine dose regimen is required to prevent nocturnal cystine accumulation in cystinosis. Pediatric Nephrol. 2005;21(1):110–113. 10.1007/S00467-005-2052-0. 10.1007/s00467-005-2052-0 16252107

[6] Fava A, Iliceto A, Camera E. Kinetics of the Thiol-Disulfide Exchange. J Am Chem Soc. 1957;79(4):833–8. 10.1021/ja01561a014.

[7] Whitesides GM, Lilburn JE, Szajewski RP. Rates of Thiol-Disulfide Interchange Reactions between Mono- and Dithiols and Ellman’s Reagent. J Org Chem. 1977;42(2):332–8. 10.1021/jo00422a034.

[8] Houk J, Whitesides GM. Structure-Reactivity Relations for Thiol-Disulfide Interchange. J Am Chem Soc. 1987;109(22):6825–36. 10.1021/ja00256a040.

[9] Theriault Y, Rabenstein DL. A Nuclear Magnetic Resonance Study of the Equilibria and Kinetics of the Reaction of Penicillamine with Cystine and Related Disulfides. Can J Chem. 1985;63(8):2225–31. 10.1139/v85-366.

[10] Singh R, Whitesides GM. Thiol—disulfide interchange. sulphur-containing functional groups (1993). John Wiley & Sons, Ltd; 1993;633–58. 10.1002/9780470034408.ch13.

[11] Beales D, Finch R, McLean AEM, Smith M, Wilson ID. Determination of Penicillamine and Other Thiols by Combined High-Performance Liquid Chromatography and Post-Column Reaction with Ellman’s Reagent: Application to Human Urine. J Chromatogr B Biomed Sci Appl. 1981;226(2):498–503. 10.1016/S0378-4347(00)86088-0. 10.1016/s0378-4347(00)86088-0 7320180

[12] Byrne FP, Jin S, Paggiola G, Petchey THM, Clark JH, Farmer TJ, Hunt AJ, Robert McElroy C, Sherwood J. Tools and Techniques for Solvent Selection: Green Solvent Selection Guides. Sustain Chem Process. 2016. 10.1186/s40508-016-0051-z.

