# Supplementary material for: A rapid HPLC method to simultaneously quantify therapeutic thiols and monitor their disulfide exchange reactions with cystine
Source: Orphanet J Rare Dis. 2026 Apr 22;21:218. doi: 10.1186/s13023-026-04363-w (PMC13277012; doi:10.1186/s13023-026-04363-w)
Supplement: Supplementary file 1 — Supplementary Material 1 [file 13023_2026_4363_MOESM1_ESM.docx]

Supplementary Information

**A rapid HPLC method to simultaneously quantify therapeutic thiols and monitor their disulfide exchange reactions with cystine**

Azhidhack Hadjipour^†^, Gayatri Gayatri^†^, Patrice Rioux^‡^ & Oisín N. Kavanagh^†^*

^†^*School of Pharmacy, Newcastle University, Newcastle upon Tyne, UK.*

^‡^*Thiogenesis Therapeutics, San Diego, California.*

*UV-Vis spectroscopy*

The absorption spectra of Ellman's reagent (DTNB) in phosphate-buffered saline (PBS) (0.1 M, pH 8.0) were examined in the presence of thiol-containing compounds, specifically cysteine and cysteamine. The experimental results demonstrate significant challenges in monitoring the generation of two distinct free thiols, primarily due to overlapping absorbance signals within the 400–410 nm wavelength range (Figure S1). It is clear that these commonly employed assay conditions are inadequate for quantifying disulfide exchange reactions.

**
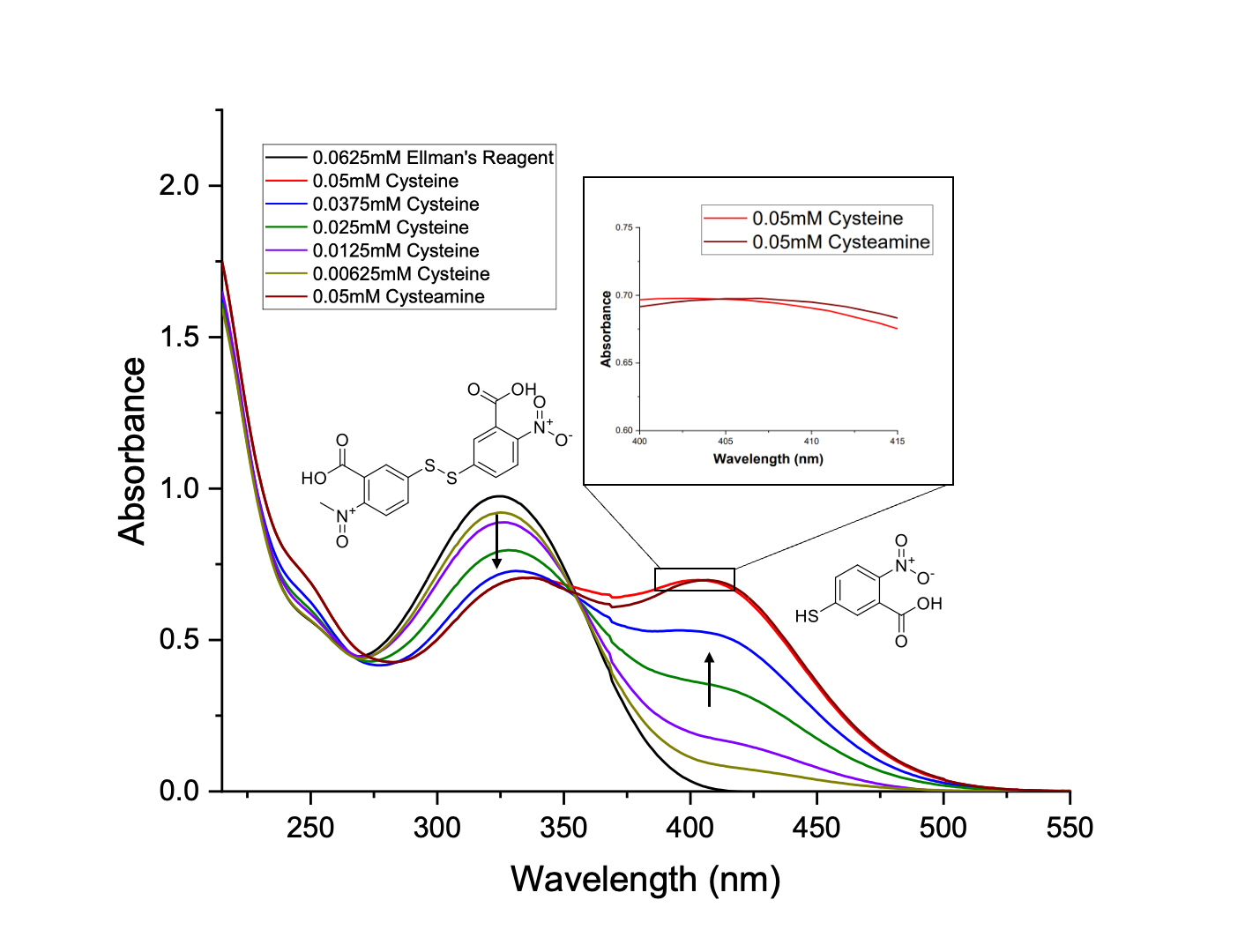
**

**Figure S1.** Ultraviolet-visible (UV-Vis) absorption spectra of Ellman’s reagent following incubation with progressively increasing concentrations of cysteine at pH 8.0 and 0.05 mM cysteamine.

**Figure S2**. A wide-spectrum analysis (200-450 nm) of a derivatised 0.05 mM cysteine sample was conducted using a DAD-equipped Agilent 1260 Infinity high performance liquid chromatography system. Based on the AUC values and their variation across different wavelengths, 248 nm was selected as the optimal wavelength for peak detection from the region with the closest range of values for all peaks.

**
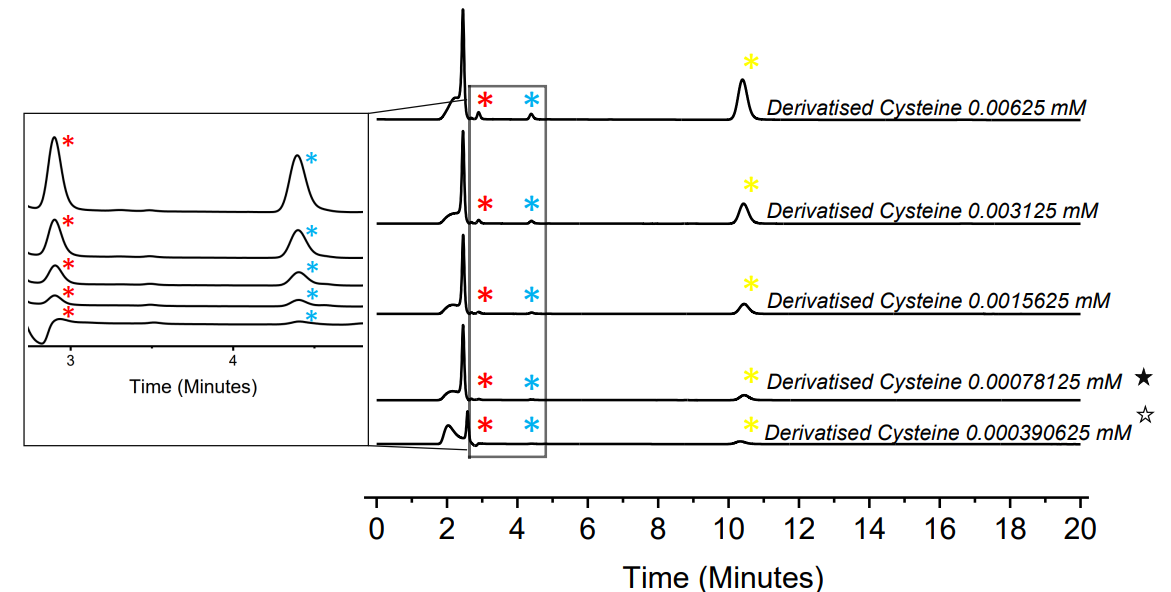
**

**Figure S3.** Stacked high performance liquid chromatography chromatograms illustrating the limits of quantification (LOQ) and detection (LOD) for derivatized cysteine samples, analysed using the optimized 40:60 (0.1% Trifluoroacetic acid: Methanol) mobile phase method. The chromatograms demonstrate the sensitivity and resolution of the method in detecting low concentrations of the analytes. Thiol-TNB adducts, 5-thio-2-nitrobenzoic acid (TNB), and Ellman’s reagent peaks are indicated by red, blue, and yellow asterisk, respectively. LOQ and LOD indicated by black and white stars, respectively.


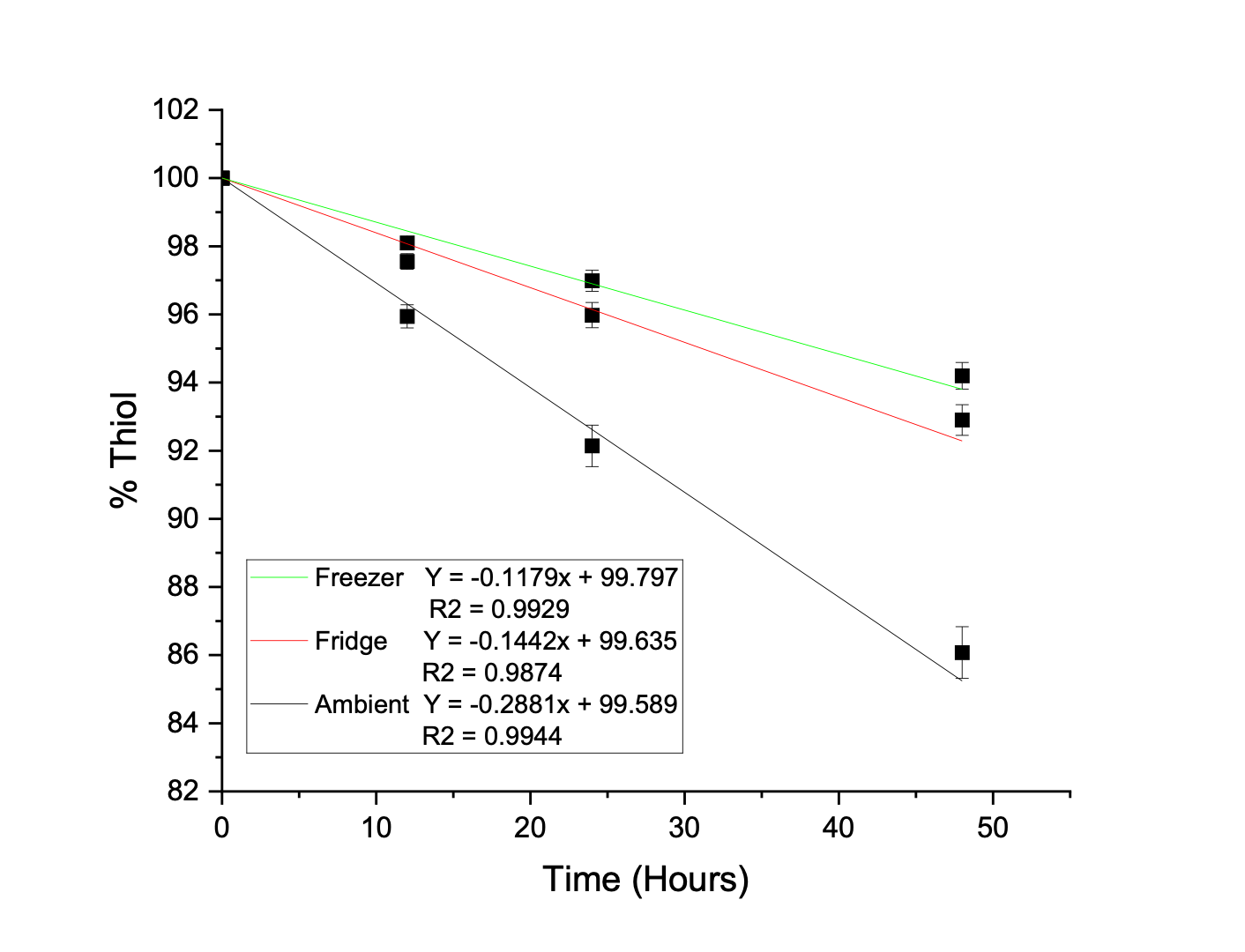

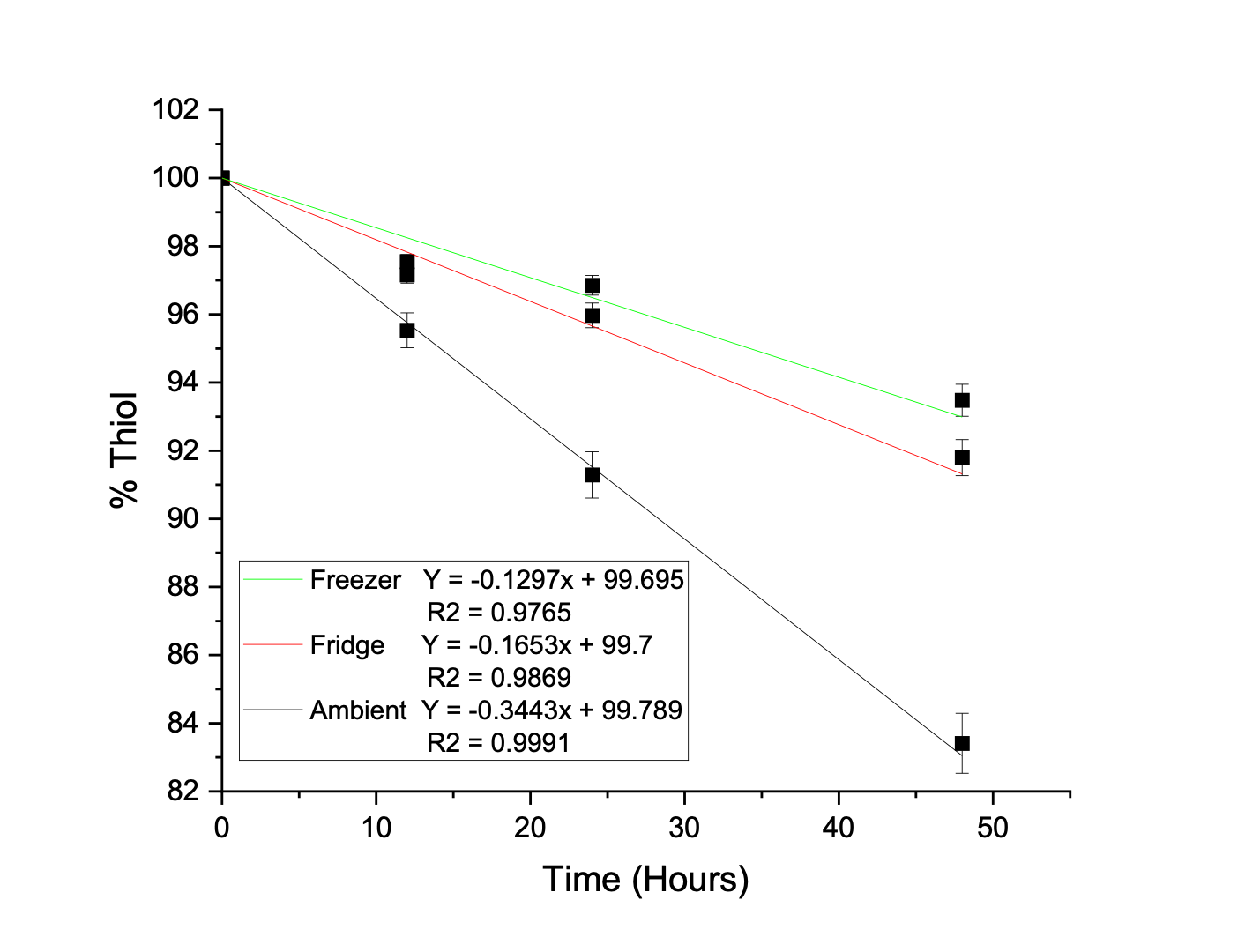


**Figure S4**. % calculated cysteine (left) and % calculated cysteamine (right) over a time period of 0-48 hours at different storage conditions. The analysis was conducted using the optimized high performance liquid chromatography method 40:60 (0.1% Trifluoroacetic acid: Methanol). Error bars represent standard deviation.

**Table S1**. Table representing free thiol quantification in phosphate-buffered saline pH 8.0, Human Saliva & Urine (vegetarian samples) as a function of thiol-TNB adduct Rt and percentage recovery. Quantification of free thiols in the Human non-vegetarian samples was not achieved due to back ground noises in the area of the interest.

|  |  | Cysteine | Cysteamine | Glutathione | Penicillamine | Tiopronin | Captopril |
| --- | --- | --- | --- | --- | --- | --- | --- |
| PBS | Rt Mean ± SD | 3.181 ± 0.010 | 3.322 ± 0.021 | 3.508 ± 0.014 | 3.699 ±  0.009 | 6.210 ± 0.009 | 12.025 ± 0.006 |
|  | Thiol (mM) | 0.067 | 0.051 | 0.052 | 0.041 | 0.049 | 0.040 |
|  | % Recovery  Mean ± SD | 134.6 ± 0.011 | 102.1 ± 0.017 | 103.1 ± 0.010 | 82.43 ±  0.002 | 97.5 ±  0.002 | 80.5 ± 0.001 |
| Human saliva  vegetarian | Rt Mean ± SD | 3.174 ± 0.007 | 3.315 ± 0.004 | 3.501 ± 0.008 | 3.692 ±  0.013 | 6.190 ± 0.058 | 11.978 ± 0.117 |
|  | Thiol (mM) | 0.058 | 0.040 | 0.044 | 0.043 | 0.049 | 0.037 |
|  | % Recovery  Mean ± SD | 116.7 ± 0.007 | 80.0 ±  0.015 | 87.2 ±  0.004 | 86.3 ±  0.001 | 97.1 ±  0.003 | 74.7 ± 0.002 |
| Human Urine  vegetarian | Rt Mean ± SD | 3.176 ± 0.003 | 3.317 ± 0.011 | 3.507± 0.003 | 3.690 ±  0.008 | 6.212 ± 0.039 | 12.053 ± 0.059 |
|  | Thiol (mM) | 0.068 | 0.043 | 0.044 | 0.048 | 0.065 | 0.040 |
|  | % Recovery  Mean ± SD | 136.3 ± 0.012 | 85.5 ±  0.006 | 88.0 ±  0.001 | 96.4 ±  0.004 | 129.2 ± 0.005 | 79.0 ± 0.004 |

*Validation of the 45:55 (0.1% TFA in water: methanol) method*

**Table S2.** Table presents the stability data for 0.05 mM cysteamine and 0.05 mM cysteine derivatised solutions, expressed as percentage concentration recovery at pre-determined time intervals. The analysis was conducted using the optimized high performance liquid chromatography method 45:55 (0.1% Trifluoroacetic acid: Methanol).

| **Cysteamine**  **0.05 mM** | **Ambient (15-25 ºC)**  **Mean ± S.D.**  **(%RSD)** | **Fridge (2 - 8 ºC)**  **Mean ± S.D.**  **(%RSD)** | **Freezer (-20 ºC)**  **Mean ± S.D.**  **(%RSD)** |
| --- | --- | --- | --- |
| T0 | 100.00 ± 0.0  (0.0) | 100.00 ± 0.0  (0.0) | 100.00 ± 0.0  (0.0) |
| T12 | 95.95 ± 0.33  (0.34) | 97.34 ± 0.12  (0.12) | 97.43 ± 0.11  (0.11) |
| T24 | 91.43 ± 0.47  (0.51) | 95.45 ± 0.21  (0.22) | 96.68 ± 0.20  (0.21) |
| T48 | 84.01 ± 0.62  (0.74) | 92.30 ± 0.32  (0.35) | 93.71 ± 0.30  (0.32) |
| **Cysteine**  **0.05 mM** | **Ambient (15-25 ºC)**  **Mean ± S.D.** | **Fridge (2 - 8 ºC)**  **Mean ± S.D.** | **Freezer (-20 ºC)**  **Mean ± S.D.** |
| T0 | 100.00 ± 0.0  (0.0) | 100.00 ± 0.0  (0.0) | 100.00 ± 0.0  (0.0) |
| T12 | 95.90 ± 0.30  (0.31) | 98.04 ± 0.09  (0.09) | 98.62 ± 0.14  (0.14) |
| T24 | 92.38 ± 0.50  (0.54) | 95.92 ± 0.19  (0.20) | 96.97 ± 0.26  (0.27) |
| T48 | 86.96 ± 0.66  (0.76) | 92.60 ± 0.22  (0.24) | 94.17 ± 0.31  (0.33) |


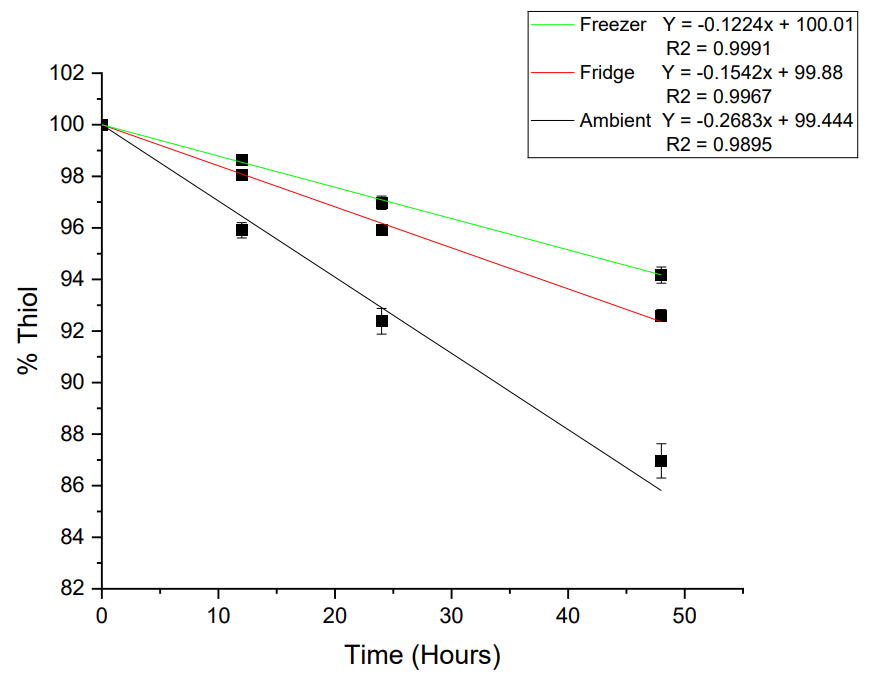

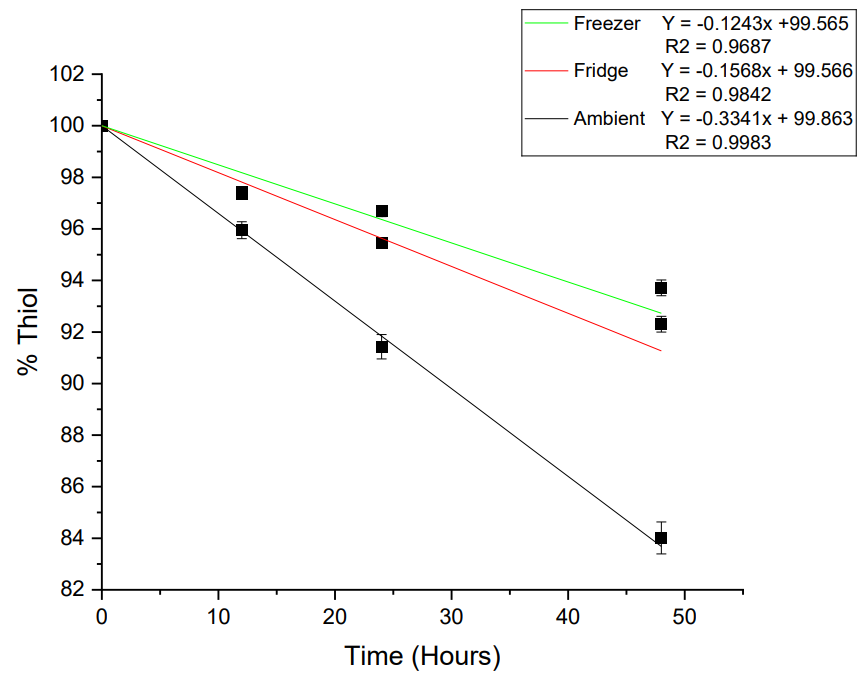


**Figure S5.** Graphs illustrate the stability data for 0.05 mM cysteamine (right) and 0.05 mM cysteine (left) derivatised solutions, expressed as percentage concentration recovery at pre-determined time intervals. The analysis was conducted using the optimized high performance liquid chromatography method 45:55 (0.1% Trifluoroacetic acid: Methanol). Error bars represent standard deviation (n=3).

**Table S3.** Table presents the percentage recovery data (Mean ± S.D.) for three independently prepared solutions of the free thiol-containing compound, cysteamine, at known concentrations. Analyses were performed using the optimized 45:55 (0.1% Trifluoroacetic acid: Methanol) high performance liquid chromatography method. The results indicate acceptable recovery rates with minimal standard deviations, thereby affirming the reliability, precision, and reproducibility of the developed analytical method.

| **Code** | **Theoretical** **(mM)** | **Obtained**  **(mM) R1** | **Obtained**  **(mM) R2** | **Obtained**  **(mM) R3** | **Mean ± S.D.** | **Recovery (%)**  **Mean ± S.D.** |
| --- | --- | --- | --- | --- | --- | --- |
| CYSI | 0.0389 | 0.0378 | 0.0375 | 0.0374 | 0.0376 ± 0.0002 | 96.57 ± 0.47 |
| CYSII | 0.0191 | 0.0188 | 0.0188 | 0.0185 | 0.0187 ± 0.0001 | 97.57 ± 0.75 |
| CYSIII | 0.0098 | 0.0094 | 0.0094 | 0.0094 | 0.0094 | 95.92 ± 0.0 |

**Table S4.** Precision analysis for both within-run and between-run variability for derivatised 0.05 mM samples of cysteamine and cysteine, using 45:55 method. The data, presented as mean ± standard deviation (S.D.) and %RSD, including the retention times of thiol-TNB adducts, retention times of 5-thio-2-nitrobenzoic acid (TNB) peaks, and concentrations of free thiols.

| **Thiol** | **TNB-adduct**  **Rt (mins)**  **Mean ± S.D.**  **(%RSD)** | **TNB**  **Rt (mins)**  **Mean ± S.D.**  **(%RSD)** | **Free Thiol Concentration (mM)**  **Mean ± S.D.**  **(%RSD)** |
| --- | --- | --- | --- |
| **Within-run** |  |  |  |
| Cysteamine | 3.39 ± 0.0024  (0.07) | 5.15 ± 0.0026  (0.05) | 0.05 ± 0.0002  (0.4) |
| Cysteine | 3.19 ± 0.0017  (0.05) | 5.15 ± 0.0026  (0.05) | 0.05 ± 0.0001  (0.2) |
| **Between-run** |  |  |  |
| Cysteamine | 3.38 ± 0.0356  (1.05) | 5.23 ± 0.0860  (1.64) | 0.05 ± 0.0005  (1.0) |
| Cysteine | 3.20 ± 0.0213  (0.67) | 5.23 ± 0.0860  (1.64) | 0.05 ± 0.0003  (0.6) |

**
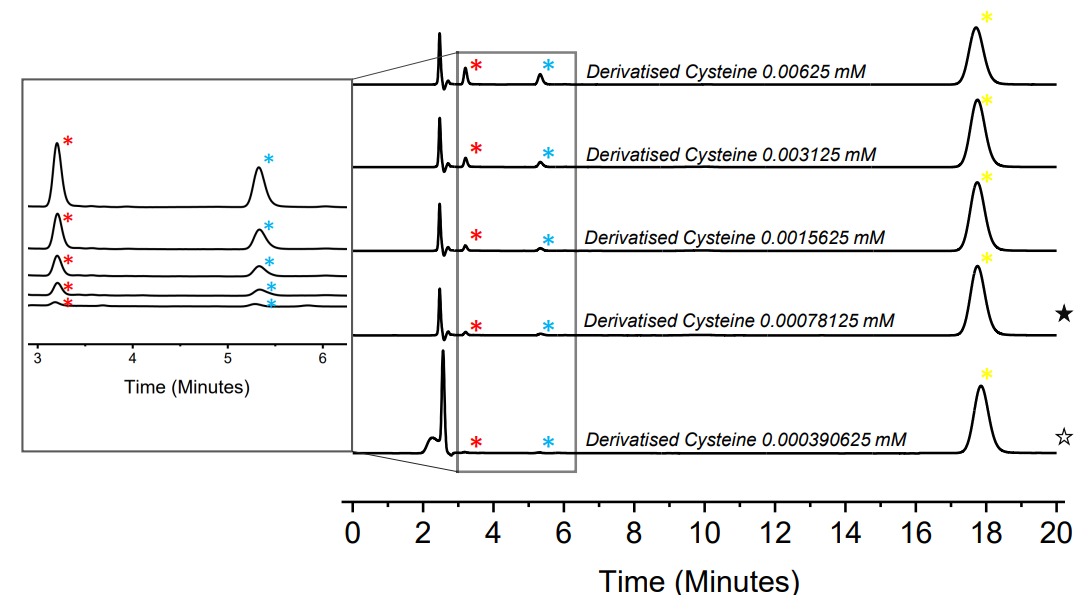
**

**Figure S6.** Stacked chromatograms illustrating the limits of quantification (LOQ) and detection (LOD) for derivatized cysteine samples, analysed using the optimized 45:55 (0.1% Trifluoroacetic acid: Methanol) method. Thiol-TNB adducts, 5-thio-2-nitrobenzoic acid (TNB), and Ellman’s reagent peaks are indicated by red, blue, and yellow asterisk, respectively. The chromatograms demonstrate the sensitivity and resolution of the method in detecting low concentrations of the analytes. LOQ and LOD are indicated by black and white stars, respectively.

**Table S5.** Limits of quantification data presented as percentage recovery (Mean ± S.D.) for derivatized cysteine and cysteamine samples. Analyses were performed using the optimized 45:55 (0.1%TFA: MeOH) method (n=6). The data reflect the sensitivity and reproducibility of the method at low analyte concentrations.

| **UV-vis** | | | |
| --- | --- | --- | --- |
| **Cysteine (mM)** | **Absorbance (A.U.)**  **Mean ± S.D.** | **Calculated Thiol (mM)** | **Recovery (%)**  **Mean ± S.D.** |
| 0.00625 | 0.0856 ± 0.0046 | 0.006244 | 99.90 ± 4.33 |
| 0.003125 | 0.0434 ± 0.0019 | 0.003167 | 101.35 ± 4.50 |
| 0.0015625 | 0.0217 ± 0.0013 | 0.001588 | 101.63 ± 6.02 |
| 0.00078125 | 0.0109 ± 0.0003 | 0.000800* | 102.40 ± 2.94 |
| **HPLC** | | | |
| **Cysteamine (mM)** | **TNB Rt (mins)**  **Mean ± S.D.** | **Calculated Thiol (mM)** | **Recovery (%)**  **Mean ± S.D.** |
| 0.00625 | 5.32 ± 0.009 | 0.00612 | 97.92 ± 2.32 |
| 0.003125 | 5.33 ± 0.009 | 0.00308 | 98.56 ± 1.40 |
| 0.0015625 | 5.33 ± 0.009 | 0.001574 | 100.74 ± 2.88 |
| 0.00078125 | 5.33 ± 0.009 | 0.000802* | 102.66 ± 1.06 |
| **Cysteine (mM)** | **TNB Rt (mins)**  **Mean ± S.D.** | **Calculated Thiol (mM)** | **Recovery (%)**  **Mean ± S.D.** |
| 0.00625 | 5.33 ± 0.009 | 0.00620 | 99.20 ± 3.19 |
| 0.003125 | 5.33 ± 0.009 | 0.003142 | 100.54 ± 1.07 |
| 0.0015625 | 5.33 ± 0.009 | 0.001575 | 100.80 ± 2.99 |
| 0.00078125 | 5.33 ± 0.009 | 0.000799* | 102.27 ± 2.60 |
| **Mean ± S.D.**  *** Limit of quantification** | | | |


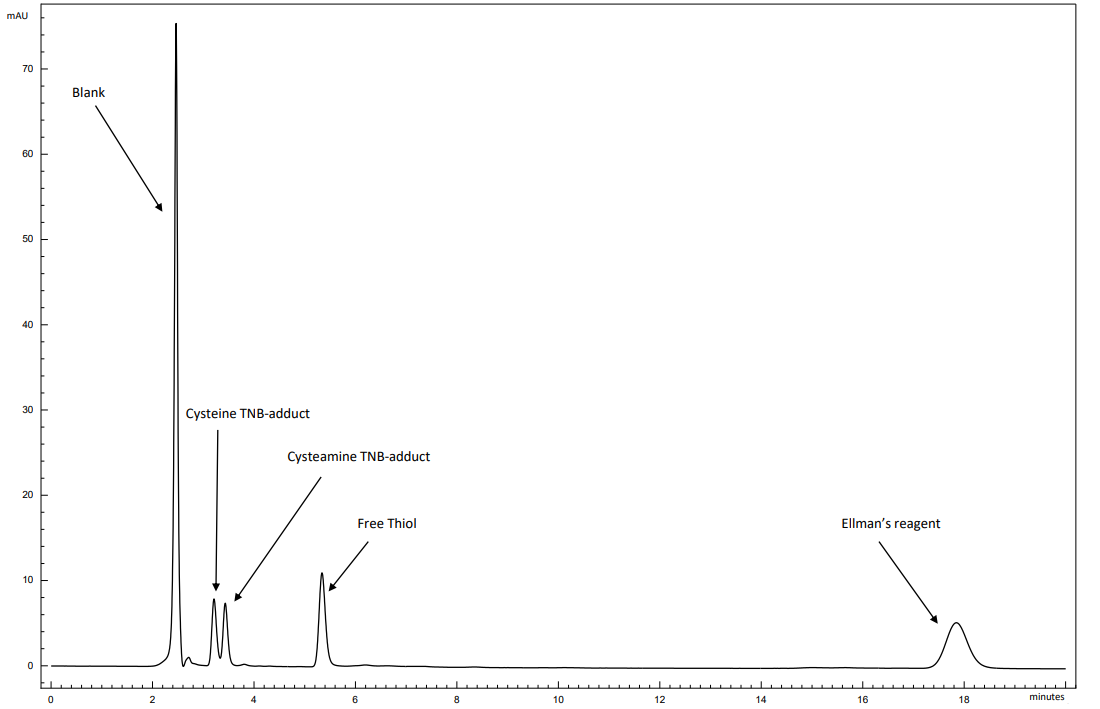


**Figure S7**. The elution profiles of the cysteine and cysteamine TNB adducts. The resolution between the thiol-TNB adduct peaks, calculated using the chromatographic resolution equation giving a resolution of 2.19, which exceeds the threshold value of 1.5, thereby indicating a satisfactory separation and supporting the reliability of the collected analytical data. As anticipated, the overlapping 5-thio-2-nitrobenzoic acid (TNB) peaks underscore the suitability of the optimized method for tracking these adducts, affirming its applicability for kinetic analysis.


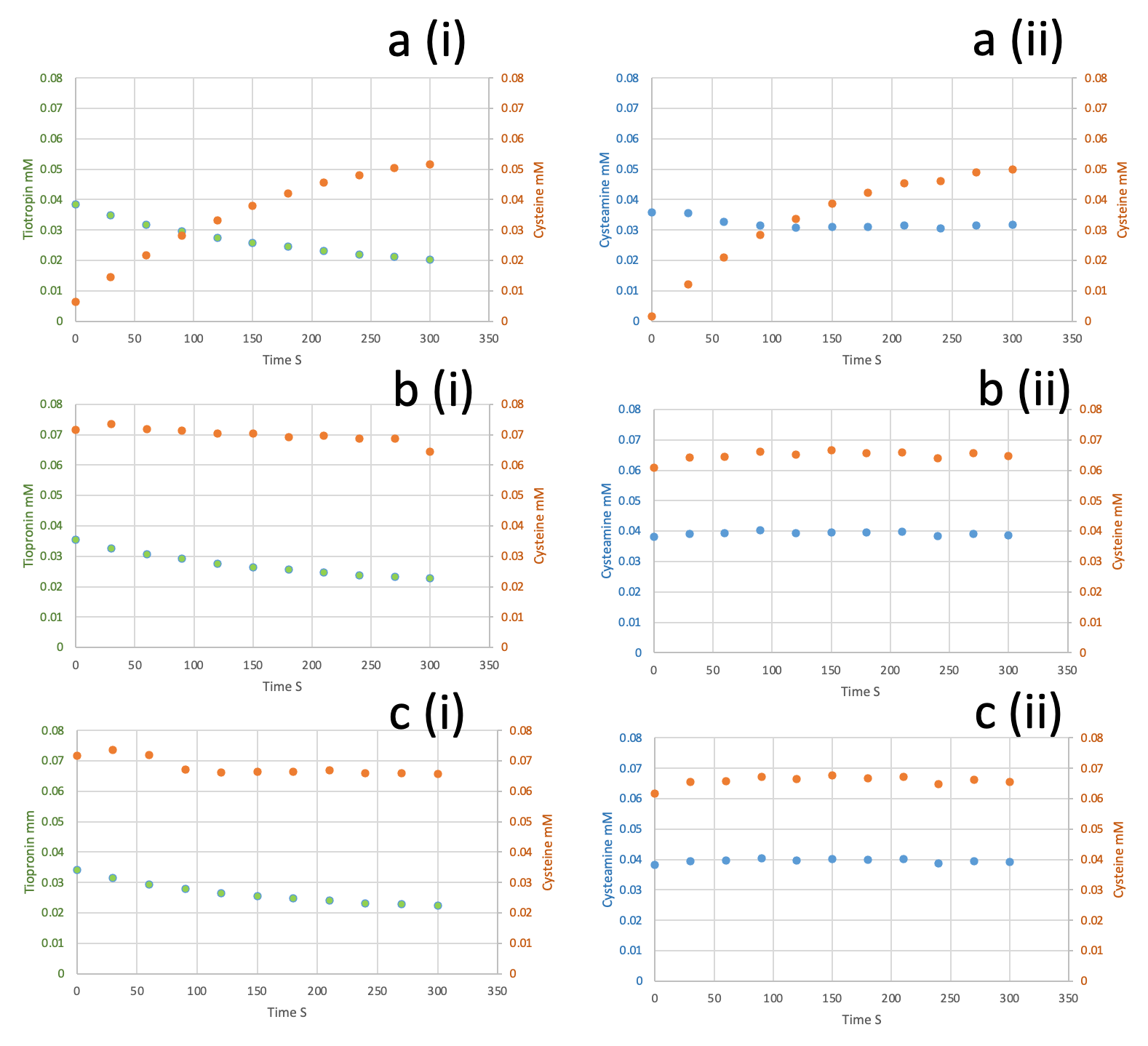


**Figure S8**. Monitoring the free thiol reaction with cystine using Ellman's reagent: a, b and c are the results of the same reaction (i = tiopronin; ii = cysteamine) injected at 30 minutes after the reaction, 24 h after the reaction and 48 h after the reaction, respectively.

**Figure S9**. Monitoring the repeated injection of a sample which illusrates this anomalous effect.
